# Phrasal Paraphrase Based Question Reformulation for Archived Question Retrieval

**DOI:** 10.1371/journal.pone.0064601

**Published:** 2013-06-21

**Authors:** Yu Zhang, Wei-Nan Zhang, Ke Lu, Rongrong Ji, Fanglin Wang, Ting Liu

**Affiliations:** 1 Research Center for Social Computing and Information Retrieval, Harbin Institute of Technology, Harbin City, Heilongjiang, China; 2 Graduate University of Chinese Academy of Sciences, Beijing City, China; 3 Department of Cognitive Science, Xiamen University, Xiamen City, Fujian, China; 4 School of Computing, National University of Singapore, Singapore, Singapore; University of Adelaide, Australia

## Abstract

Lexical gap in cQA search, resulted by the variability of languages, has been recognized as an important and widespread phenomenon. To address the problem, this paper presents a question reformulation scheme to enhance the question retrieval model by fully exploring the intelligence of paraphrase in phrase-level. It compensates for the existing paraphrasing research in a suitable granularity, which either falls into fine-grained lexical-level or coarse-grained sentence-level. Given a question in natural language, our scheme first detects the involved key-phrases by jointly integrating the corpus-dependent knowledge and question-aware cues. Next, it automatically extracts the paraphrases for each identified key-phrase utilizing multiple online translation engines, and then selects the most relevant reformulations from a large group of question rewrites, which is formed by full permutation and combination of the generated paraphrases. Extensive evaluations on a real world data set demonstrate that our model is able to characterize the complex questions and achieves promising performance as compared to the state-of-the-art methods.

## Introduction

As the blooming of Web 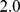, community question answering services (cQA) have emerged as popular means for knowledge dissemination and information seeking, such as Yahoo! Answers, WikiAnswer and Quora, etc. Over times, an overwhelming amount of QA pairs with high quality devoted by human intelligence has been accumulated as comprehensive knowledge base, which greatly facilitates users to seek precise information by querying in natural language, rather than issuing the key words and painstakingly browsing through large ranked lists of results in order to look for the correct answers.

However, question retrieval is nontrivial. One major reason is the lexical gap between the queried questions and the archived historical questions in repositories. This is due to the variability of languages, which directly leads to both of the content contributors and seekers conveying their intentions in different word forms, even describing the same meanings. As shown in Table 1, Q1 and Q2 are semantically similar but lexically different questions.

**Table 1:** Representative questions for lexical gap illustration.

**Query:**
Q1: Can you catch a cold from cold temperature?
**Expected:**
Q2: Does cold weather affect actually catching a cold?
**Not Expected:**
Q3: How can you catch a cold?
Q4: Can you catch a cold from getting your head wet?

Paraphrasing techniques can gracefully bridge the lexical gap in question retrieval. As described in [1], paraphrases are alternative ways of conveying the same information. For example, the phrases “catch a cold” and “get colds” are paraphrases as well as the phrases “expectant mother” and “pregnant”. The existing approaches generally fall into two categories according to different granularities. One is lexical-level [2]–[4], aiming to acquire word level paraphrases by extracting the synonyms from dictionaries or monolingual and bilingual corpora. The other is sentence-level, extracting semantically similar questions from query log or web search results [5], [6]. However, the former fails to characterize the relations among terms. It means that the words are independent with their adjacent context. The latter still faces the challenges that are not easy to tackle, such as the deep understanding of the complex questions with sophisticated syntactic and semantic, and taking the context into consideration to generate the equivalent candidates. Therefore, new approaches towards paraphrasing questions in appropriate level of granularity are highly desired.

**Figure 1:**
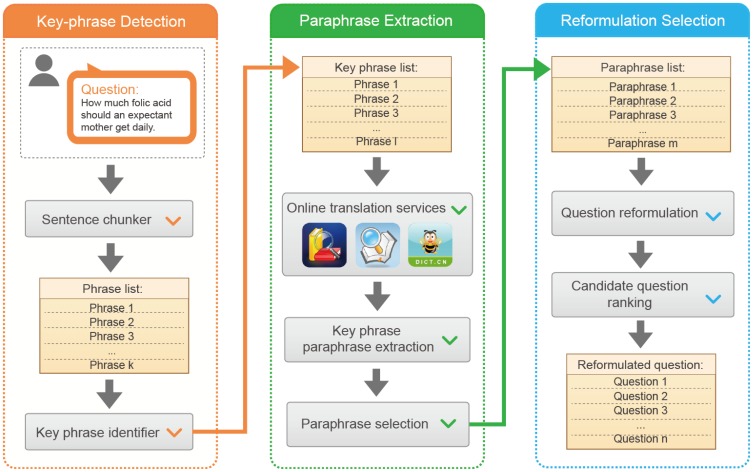
The schematic illustration of the proposed question reformulation scheme.

In this study, we propose a question reformulation scheme that makes an intelligent use of paraphrases in phrase-level of the queried questions. It is able to increase the likelihood of finding the relevant or similar questions which are well answered or voted by other users. As shown in Figure 1, the scheme consists of three components. Given a question, the first component detects the involved key-phrases by jointly exploring the corpus-dependent knowledge and question-aware cues. Second, it automatically paraphrases each identified key-phrase utilizing multiple online translation engines. The third component selects the most relevant reformulations from a large group of question rewrites, which is formed by full permutation and combination of the generated paraphrases. By conducting extensive experiments on a large data set, we demonstrate that our proposed scheme yields significant gains in question retrieval performance and remarkably outperforms other state-of-the-art technologies.

The remainder of this paper is organized as follows. The Related Work section briefly reviews the related work. In Sections of the Key-Phrase Detection and Proposed Phrasal Paraphrase Extraction, we introduce the key-phrase identification and paraphrase-candidates generation, respectively. We then describe the paraphrase selection and question reformulation approach in Section Question Reformulation Generation. Experimental results and analysis are presented in Section Experimental Results, followed by the conclusion and future work in the last Section.

## Related Work

### Key Phrase Detection

Question sentences in cQA are usually surrounded by various description sentences, and expressed by informal languages such as question mark etc. Key phrase detection is important for not only QA but also other tasks, such as tag-based image retrieval [7], [8], tweet summarization [9], and social media analysis [10]–[12]. In 2009, Wang et al. [13] proposed a syntactic tree matching model to find similar questions, which was demonstrated to be robust against grammatical errors. One year later, they [14] exploited salient patterns to improve question retrieval results. Bendersky et al. [15] captured the lexical, statistical and n-grams features to identify key concepts (phrases) from verbose queries. Later, [16] proposed a learning method to estimate concept weights in verbose queries using Markov random field model. Then they parameterized concept weights and integrated the weights into query expansion model [17]. Recently, they modeled concepts dependencies in queries using hypergraphs [18]. However, these work only focuses on distinguishing key concepts from other non-key concepts and the differences between key concepts are not considered. In this paper, we propose a ranking based method to tackle the problem and improve the performance of key concept detection approach.

### Question Term Weighting

The queries are usually depicted in form of natural languages including various sophisticated syntactic and semantic features, rather than the simple key words supported by current dominant web search engines. Therefore, one of the major challenges is how to capture the syntactic and semantic relations among query terms. Song et al. [19] and Srikanth et al. [20] shifted from the unigram to bigram and bi-term in language model to capture the term dependence in queries. An advanced dependency language model was proposed in [21], which exploited term relations using dependency parsing and integrated the dependency relations into the traditional language model. Further, Cui et al. [22] tried to capture the similarity between different dependency relation paths of the two same terms using translation model.

To compare with other indexed documents, the archived questions in current cQA forums are usually very short, which are hard to be matched by conventional lexicon and statistics based approaches. Hence, the research community in cQA retrieval faced the challenge of question expansion. Some researchers utilized the lexical dictionary, such as WordNet, to fuzzily match the synonyms between the queried questions and the reservoirs [2], [3], [23]. Some others [1], [24], [25] employed the term co-occurrence to expand queries and the translation model to estimate the semantic similarities among words.

### Pivot Paraphrasing Approach

The diversity of expressions in natural language for the similar questions directly results in mismatch in the question retrieval task. This is the so-called lexical gap. Word-level [2]–[4] and sentence-level [5], [6] question paraphrasing technologies have been proposed to relieve this problem, but obtained not very satisfying results.

### Key-Phrase Detection

**Figure 2:**
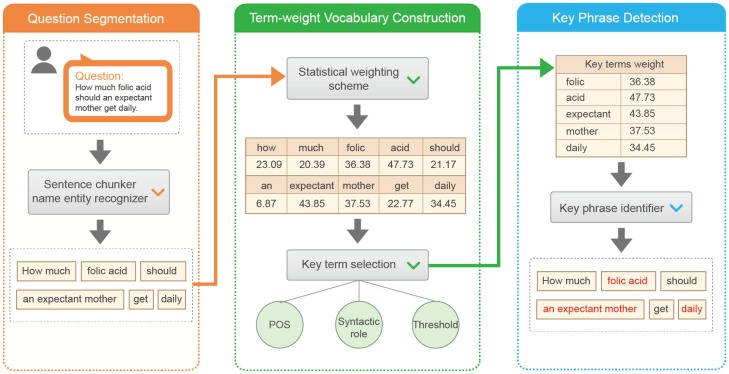
An illustration of key-phrases detection from a given question in natural languages.

A key-phrase in a given question is generally one or more words constituting an indispensable part of the question meaning. Beyond key-phrases, questions tend to contain several redundant chunks, which have grammatical meanings for communication among humans to help understanding users' intents. However, they are almost useless in describing the key concepts. For instance, for the question “How much folic acid should an expectant mother get daily?”, the two phrases “folic acid” and “expectant mother” essentially express the meaning of the original question and “daily” is temporal expression. They play the important roles in question match. The use of other chunks may bring unpredictable noise to question retrieval. Therefore, to distinct the key-phases from others, we propose a heuristic framework as illustrated in Figure 2.

The core part in this framework is an automatically constructed term-weight pair vocabulary. Given a question, we extract its constituent key-phrases as follows:

1. We segment the given question into chunks using the openNLP (http://incubator.apache.org/opennlp/) toolkit.
2. We construct a vocabulary containing term-weight pairs by leveraging the corpus-dependent knowledge. Specifically, we archive all the terms selected from our data set and their estimated weights (to be introduced in Section Question Terms Weighting).
3. For each chunk, we fetch each term weight from our constructed vocabulary. If one of its term weights satisfies the question-dependent constraints (to be introduced in Section Question-aware Constraints), the chunk is classified as a key-phrase. The detected key-phrase is used as the basic unit for paraphrasing.

### Question Terms Weighting

In this paper, we quantify question terms using the following equation inspired by BM25 [26] and LM [27],
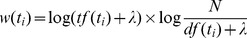
(1)where 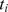 represents the 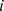-th term in question, 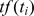 and 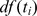 represent term frequency and document frequency, respectively. In question retrieval task, a document usually indicates a candidate question for retrieval. 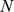 is the total number of questions in our corpus. 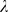 is a smoothing parameter. It is observed that term weight is independent of a specific question, but aware of corpus knowledge. Later, we will present the term weighting based key-phrase detection approach.

### Question-aware Constraints

Actually, we regard the key-phrase detection task as a key term identification problem. To be specific, if a chunk contains a key term, it will be classified as a key-phrase. Meanwhile, we predict whether a term is a key term by exploring multi-faceted cues, such as the part-of-speech (POS) of the term, as well as the term syntactic role in the question dependency parsing tree. Here, we use the Stranford CoreNLP [28] toolkit to get the POS and the syntactic roles of terms.

Given a question 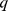, we employ three question-dependent thresholds to capture the average weights inside of 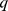 as follows.

The first one is the quadratic mean value based weight. Quadratic mean value of a set of values is the square root of the arithmetic mean (average) of the squares of the original values. Here, we represent the quadratic mean value as 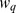 which can be calculated as follows:
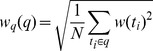
(2)where 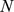 is the question length on words. 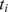 represents the 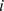-th term in 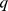, and 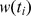 represents the weight of 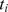, which is computed by Equation 1. The second threshold is the arithmetic mean weight which is represented as 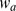 and computed as follows:
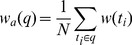
(3)The arithmetic mean is the central tendency of a collection of numbers taken as the sum of the numbers divided by the size of the collection.

The third threshold is geometric mean weight which is represented as 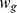 and computed as follows:
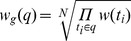
(4)The geometric mean, in mathematics, is a type of mean or average, which indicates the central tendency or typical value of a set of numbers.

As the weight of each term is positive and when question 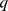 is given, there exists a relationship among the above three thresholds in mathematics as follow, the relationship may be special when question length equals to 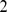. Actually, the average question length in our data set equals to 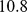 and even the shortest question still contains 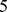 words.
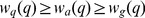
(5)Then, we use a heuristic method to identify the key terms.

In Table 2, 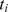 represents the 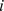-th term in question 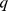. 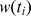 indicates the weight of term 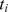; 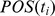 indicates the POS of term 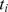; 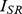 indicates the set of important syntactic role. Here, we empirically define the “nsubj” and “dobj” as the important syntactic roles, where in Stanford CoreNLP toolkit, dependency parsing labels “nsubj” and “dobj” represent the noun subject and object of predicate respectively.

**Table 2:** The heuristic method for key-phrase detection.

Key-term Decision Rules	Result
*w*(*t_i_*)≥*w_q_*(*q*)	True
*POS*(*t_i_*) = = VB and *I_SR_*(*t_i_*) = = True and *w*(*t_i_*)≥*w_a_*(*q*)	True
*POS*(*t_i_*) = = NN and *I_SR_*(*t_i_*) = = True and *w*(*t_i_*)≥*w_g_*(*q*)	True
Otherwise	False

In Table 2, the three thresholds are used as the question-aware constraints for key term identification. Given a query question and its original term weights, these thresholds can indicate the average term importance inside of the question in three measurements. By using the average term importance, we actually want to capture the different distributions of the term weights and select the salient terms as the key terms. As the three measurements have different scales, we add the external information, which is the POS and syntactic role, to distinguish the key and non-key terms.

Here, VB and NN represent two POS sets respectively. For instance, VB contains the POS of VBP, VBZ, VBG etc. NN has similar conditions with VB. As the relationship of 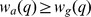 exists and NN has been proven to be more reliable [15], [29], [30] in information retrieval, we empirically consider that NN is more important than VB as the decision rules representing. As there exists the relation in 5 and the different importance between NN and VB, we set the priority of the decision rules as shown in Table 2.

The term weight threshold design is inspired by [31], [32], and this approach is widely used in [33], [34]. In addition, there are many research efforts focused on key term selection, ranking and weighting in document and other scenes, such as [25], [35], [36]. Although the three thresholds are designed heuristically, they are based on the intra-question features which are depended on the average term weights inside of a question. Hence, they are robust to the key term detection task.

**Figure 3:**
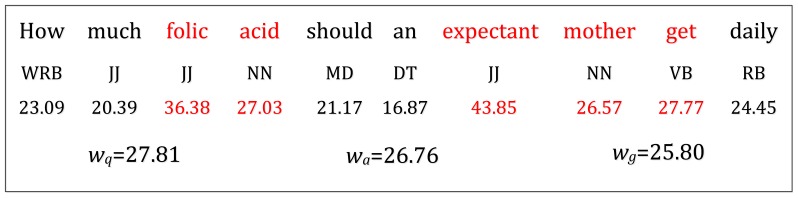
An example for illustrating term weighting scheme and key term selection.

Figure 3 shows an example of key term detection result of question “How much folic acid should an expectant mother get daily?”. The words in red color are viewed as key terms, followed by the POS tags, terms' weights and the values of three thresholds.

### Proposed Phrasal Paraphrase Extraction

In this section, we introduce our proposed online translation engine-based approach to paraphrasing each identified key-phrase from a given question. The original idea of paraphrase extraction is machine translation process within monolingual [37], [38] and bilingual [1], [39]–[41] parallel corpora. We extend the state-of-the-art technology by using online translation engines as pivot language generators. Specifically, we first translate the detected key-phrase 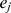 into pivot language phrase 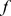 in the form of intermediate language through multiple online translation engines, such as iciba (http://www.iciba.com/), youdao (http://dict.youdao.com/) and dict (http://dict.cn/). Here, we only use Chinese as pivot language, multi-pivot languages can be used in our future work for paraphrase extraction. We then translate 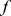 back to English and obtain the paraphrases of the original detected key-phrase. Formally, we estimate the paraphrase probability between the key-phrase and its corresponding paraphrase as:
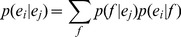
(6)where 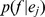 represents the probability of the original language phrase 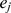 translated into the pivot language phrase 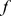. While 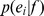 stands for the translation probability of paraphrase candidate 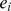 given 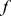. These two conditional probabilities can be estimated in a unified form as:
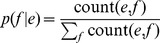
(7)To make the paraphrase model more robust, we integrate it with the language model and restate it as:
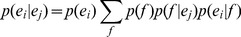
(8)

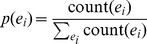
(9)It can be intuitively interpreted as follows: the probability of the phrase 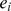 is the ratio of its frequency to the sum of all the phrases frequency. Where, 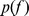 can be estimated in the same way as 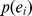.

Here, we present an instance to show how our paraphrases generation component works. For the detected key-phrase “expectant m other”, we first send it to the three dictionary systems as a query, and then we collect its translations, dictionary explanations and the network interpretations. According to our statistics, “
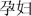
” appeared 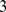 times and “
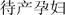
” appeared 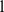 time. Following that, we translated these Chinese phrases back to English. Taking “
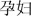
” as an example, we obtained “expectant mother”, “pregnant mother” and “gravida” 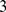 times, respectively. Therefore, count(“
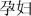
”, “expectant mother”) = 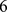, count(“
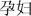
”, “gravida”) = 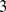.

### Question Reformulation Generation

**Figure 4:**
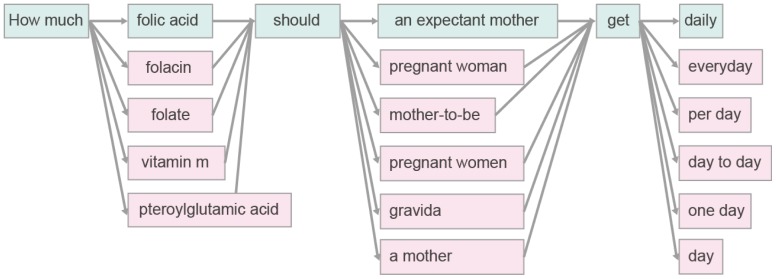
Illustration of question reformulations in the form of Viterbi decoding structure.

As shown in Figure 4, for the question “How much folic acid should an expectant mother get daily?”, 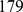 question reformulating candidates are generated with shuffling of the paraphrases in phrase level. However, some rewrites may drift away from the original meaning or not satisfy the common ways of language expression, such as this candidate “How much folate should a mother get daily”.

In this paper, we seamlessly integrate the Viterbi algorithm [42] with our proposed language model to filter out the “incorrect” reformulations. The Viterbi algorithm is a dynamic programming algorithm for finding the most likely sequence of hidden states, which is called Viterbi path, that results in a sequence of observed events. Before introducing our approach, we first define some notations. For a given question 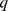, 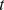 denotes the detected key-phrase position starting from 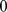. And Syns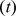 is its corresponding paraphrase set. 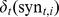 is the Viterbi variables meaning the maximum sentence probability from the beginning of the question to the current 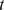-th key-phrase, which is replaced with 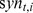
(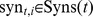). The Viterbi value is formally defined as:
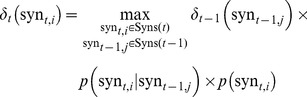
(10)where 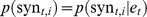 which can be estimated by Equation 8, 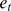 is the 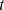-th key-phrase in question 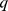.

To estimate the conditional probability 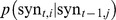, a novel bi-gram language model in phrase level is proposed by exploiting the external knowledge, Google N-gram, which was extracted from 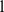
T web corpus (http://www.ldc.upenn.edu/Catalog/CatalogEntry.jsp?catalogId=LDC2006T13). To be specific, supposing 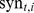 and 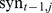 are segmented into the word sequences 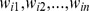 and 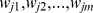 respectively. The conditional probability 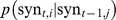 can be formulated as:
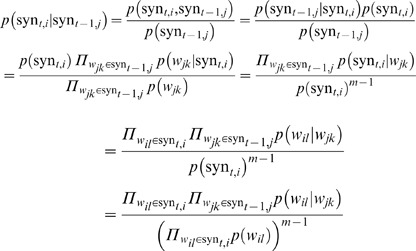
(11)where 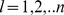 and 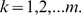. Meanwhile, 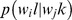 is estimated by Equation 12 with add-delta(Lidstone's law) smoothing, where 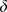 is the smoothing parameter and 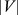 is the size of vocabulary.
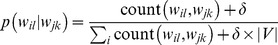
(12)Now, we have a ranked list of question reformulations for each queried question.

At last, we get the 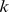 best reformulated questions as the final results. We will present how to fix 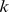 in Section Experimental Results.

**Figure 5:**
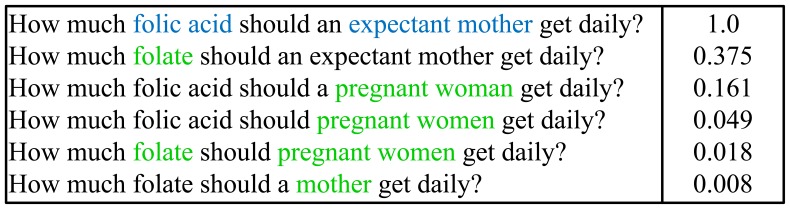
Top 5 reformulated questions with their generation probabilities.

For instance, “How much folic acid should an expectant mother get daily?”, the top five reformulated questions of the original question are shown in the following Figure 5.

## Experimental Results

### Data Set

We collected a large real world data set from Yahoo! Answers, which contains 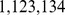 unique questions as our searching corpora and covering a wide range of topics, including health, internet, etc. We randomly selected 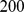 questions from this collection as our searching queries. We processed these queries by manually filtering ill-formed questions, ungrammatical or incomplete ones. After preprocessing, we obtained 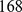 questions. From the remaining questions, we randomly chose 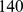 questions for testing, and the rest 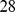 questions were used for development. The experimental data is available at http://pan.baidu.com/share/link?shareid=343582&uk=2903372971.

### On Paraphrase Extraction

We employ manual judgement, which is used in [1], [39], to evaluate the phrasal paraphrase extraction method. Two native English speakers are involved in the processing to produce their judgements as to whether the extracted paraphrases are in the same meaning with the original phrases and grammatically correct. We set 20% of overlapped data to compute their agreements and get 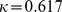, which is interpreted as “good” agreement. Here the kappa coefficient is a statistical measure of inter-annotator agreement for categorical items.

For performance comparison, we introduce the state-of-the-art method on phrasal paraphrase extraction, which is proposed in [39]. The toolkit which is published by Callison-Burch et al. can be found in http://www.cs.jhu.edu/~ccb/howto-extract-paraphrases.html, as our baseline on paraphrase extraction. In this method, the authors improved the classical pivot language translation approach to extract phrasal paraphrases from bilingual parallel corpora [1] by using syntactic constraints.

After phrase extraction from the 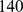 original testing queries, we obtain a total number of 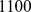 phrases. For the baseline method, we empirically set a threshold of paraphrase probabilities, which are computed using the approach in [39], to filter the paraphrases of lower quality. Finally, we obtain 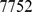 paraphrases for the original phrases.

The experimental results are shown in Table 3 with the evaluation of correct meaning (CM), correct grammar (CG) and both correct (BC) in 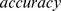. Meanwhile, we also check the macro-average and micro-average of CM, CG and BC. Here, macro-average means that we compute the average score of the paraphrase generation results for all the phrases. And micro-average represents that we compute the paraphrase generation results for each phrase respectively, and then calculate the average score of these results. All of the results are statistically significant over the respective baseline at 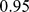 confidence interval using the 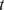-test. Here, the 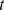-test works for testing the statistical significance on paraphrase generation result which contains a large number of 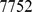 paraphrases. As the 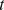-test works for the non-normal data only if the sample size is large, the 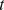-test used in our experimental data set is rational. %chg denotes the performance improvement in percent of our method over the corresponding baseline.

**Table 3:** Experimental results of phrasal paraphrase extraction both on percentage of correct meaning and grammar.

	macro-CM	micro-CM	macro-CG	micro-CG	macro-BC	micro-BC
Baseline	0.4724	0.5575	0.7825	0.8090	0.4617	0.5375
%chg	+73.05%	+65.33%	+7.13%	+18.89%	+76.87%	+68.02%
Our Method	0.8175	0.9217	0.8383	0.9618	0.8166	0.9031

From Table 3, we can see that our method outperforms the baseline method, which is the state-of-the-art method on paraphrase extraction using bilingual parallel corpora. For the experimental results, we have the following analysis.

First, the effect of baseline method for paraphrase extraction is mainly depended on the quality of word alignment in corpora. Hence, bad quality of word alignments may cause worse results in paraphrase extraction, especially on fixing the boundary of phrases.

Second, although the baseline method used syntactic match as a constraint for paraphrase extraction, there are many phrases or fragments, which have different POS and tense with original phrases, are extracted as paraphrases. It usually causes the grammar error on paraphrase extraction.

Different from the state-of-the-art method, the proposed method uses online translation engines to translate the original phrases into pivot language, which is Chinese, and then translates these pivot phrases back to the original language. The online translation engines work better on boundary control for phrases and contain the information from high quality semantic dictionaries, meanwhile, they automatically transfer phrases in different forms and tense into uniform expressions, therefore, the proposed method works better on paraphrase extraction.

One limitation is that the quantity of extracted paraphrases is less than the baseline method. For the 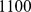 phrases, we only obtain 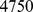 candidate paraphrases. However, the quality of paraphrases extracted by our method is higher than baseline. In future work, we plan to combine the two methods to get better performance on paraphrase extraction.

### On Question Reformulation

As the previous description, this section we will check the effectiveness of our question reformulation method for question retrieval task.

For performance comparison, we introduce two methods as the baselines for question reformulation. The first one is synonym based question reformulation which is selected as the baseline-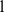 in our experiments. Here, we use WordNet as the lexical resource and distance based word similarities are used for synonym selection. The second one is the state-of-the-art method on sentence-level paraphrase generation, namely statistical paraphrase generation (SPG) [43]. Here, we consider that the generated question paraphrases can be seen as the reformulated questions. Therefore, we run SPG under the setting of baseline-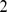 as described in [43].

Then, we utilize the three kinds of reformulated questions as queries for the question retrieval model. Hence, we can get the performance comparisons among these methods. Here, we use the state-of-the-art question retrieval model which is proposed by Xue et al. [44], namely translation-based language model (TLM), as our basic question retrieval unit. Thus, we get three question retrieval systems, the first is WordNet-based question reformulation TLM (WN-TLM), the second is SPG-based question reformulation TLM (SPG-TLM) and the last is our proposed Viterbi decoding-based question reformulation TLM (VD-TLM).

As the reformulated questions can be seen as the rewriting of original question queries, we design the performance comparisons between question retrieval systems which only use the reformulated questions as queries and the systems that use both original and reformulated questions as queries. For the latter one, we need to combine and re-rank the question retrieval results. Here, we use the blending model which is proposed by Xu et al. [45], to complete the retrieval results combination and re-ranking. It considers the reformulated questions (rq) as the similar queries to the original questions (oq), and then we capture the four kinds of similarities which are the similarity between oq and rq, the similarity between oq and rq retrieval result, the similarity between rq and oq retrieval result, the last similarity between oq retrieval result and rq retrieval result. Finally, we use linear combination to joint these similarities for question retrieval results re-ranking.

For evaluation of question retrieval, we use precision at position 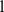 (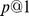), mean average precision (MAP) and mean reciprocal rank (MRR). We pool the top 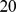 results from various methods, such as vector space model, okapi BM25 model, language model and our proposed methods, to establish our ground truth with manually checking. We asked two annotators serve their judgements on whether each of the retrieved question is similar or relevant (score 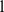) with the original question or not (score 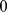). When conflicts occur, a third annotator was involved in making the final decision.

The parameters in TLM are well tuned on the development set using grid search. The experimental results on question retrieval are shown in Table 4.

**Table 4:** Overall performance comparison of MRR, MAP and p1. All improvements obtained by VD-TLM are statistically significant over other methods within 0.95 confidence interval using the 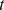-test.

Question Retrieval Models	TLM	WN-TLM	SPG-TLM	VD-TLM	WN-TLM	SPG-TLM	VD-TLM
	(oq)	(rq)	(rq)	(rq)	(oq+rq)	(oq+rq)	(oq+rq)
MRR	0.1889	0.1875	0.2024	0.2157	0.2206	0.2301	0.2583
% MRR improvement over TLM	N/A	N/A	+7.15	+14.19	+16.78	+21.81	+36.74
WN-TLM(*rq*)	+0.75	N/A	+7.95	+15.04	+17.65	+22.72	+37.76
SPG-TLM(*rq*)	N/A	N/A	N/A	+6.57	+8.99	+13.69	+27.62
WN-TLM(*oq*+*rq*)	N/A	N/A	N/A	N/A	N/A	+4.31	+17.09
SPG-TLM(*oq*+*rq*)	N/A	N/A	N/A	N/A	N/A	N/A	+12.26
MAP	0.2889	0.2870	0.3037	0.3269	0.3384	0.3664	0.4188
p@1	0.1928	0.1967	0.2214	0.2357	0.2429	0.2643	0.2786

From Table 4, we draw the following observations:

First, comparing to the state-of-the-art question retrieval model, TLM, all of the reformulation-based methods outperform TLM except WN-TLM on MRR, which indicates that question reformulation is necessary and effective for question retrieval. The reason may be that word mismatch problem is widely existing between the queries and the candidate questions. Hence, effective question reformulation methods lead to better retrieval performance as they can bridge the lexical gap. The performance of WN-TLM on MRR is a bit lower than TLM, which indicates that the synonym expanded by WordNet is not always available to real world data, some expanded words may cause chaos in meaning.

Second, both the experimental results on rq and oq+rq indicate that our proposed VD-based question reformulation methods outperform the WN-based and SPG-based methods under the results of question retrieval. It proves that VD-based question reformulation methods are more effective on question reformulation. For the WN-based question reformulation methods, they only capture word level synonyms and overlook the importance of context information. The SPG-based question reformulation methods only focus on generating high quality question paraphrases and overlook the statistical distribution of the phrases in the whole corpus. Hence the generated paraphrases may not well adapt to the archived question retrieval.

Third, the oq+rq-based methods correspondingly outperform the rq-based methods which indicates that the retrieval results which only use rq as queries could lead improvements over TLM method. However, oq cannot be overlooked, as the strict word match is also important for question retrieval. It means that question retrieval results, which use oq as queries, can be enhanced by combining rq results, through the using of the blending model for re-ranking.

### Performance Variation to the Number of Reformulated Questions

**Figure 6:**
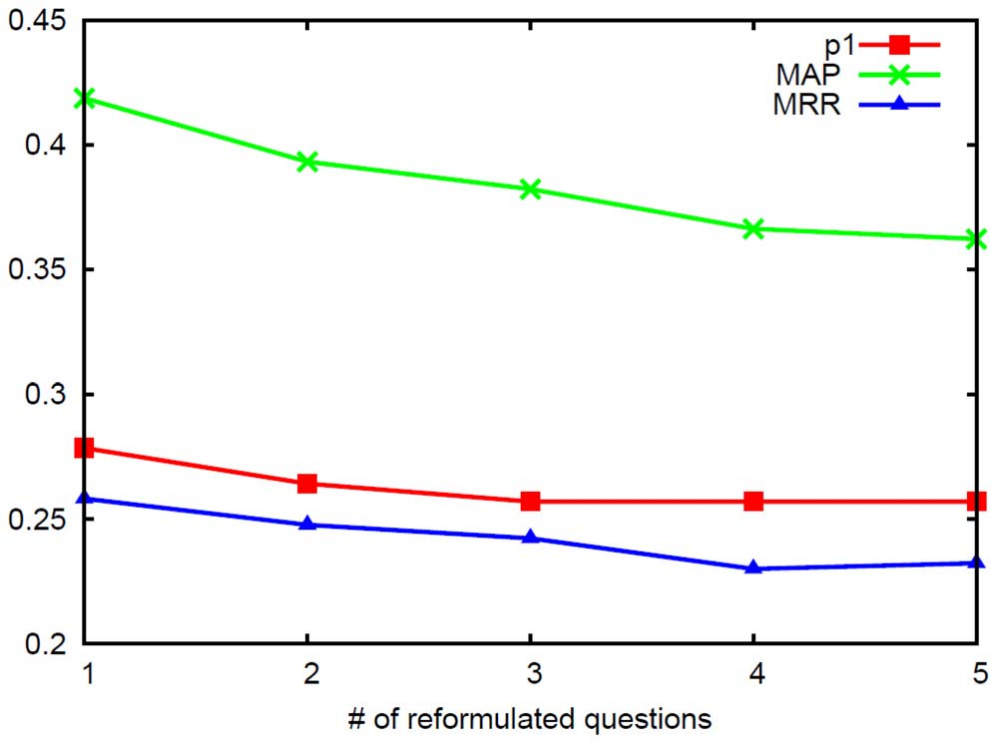
Illustration of performance variation when different number of reformulated questions are added in blending model for question retrieval.

In this section, we will check how the number of reformulated questions adding to the question retrieval model, influences the performance of the question retrieval results. Hence, we generate the top 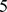 question reformulation results to observe the performance variation. Here, we use the VD-TLM model for the question retrieval task. Each time, we add the retrieval results of one reformulated question into the blending model and obtain the results in 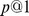, MAP and MRR. Finally, we draw the performance variation in Figure 6.

From Figure 6, we can see that the best performance can be achieved when the number of reformulated questions equals to 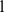. And then the performance decreases with the number growing. This indicates that better performance can be achieved by only choosing 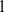 reformulated question for the retrieval results blending. This is because more reformulated questions may introduce more noise for question retrieval. Meanwhile, the query intent may be shifted by adopting more than 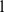 reformulated questions. Hence, in the question retrieval systems, more reformulation queries may lead to more noise.

## Conclusion and Future Work

In this paper, we presented a novel question reformulation method for archived question retrieval. Given a question, we first automatically extracted key-phrases and employed three online translation engines to obtain their translations. Further we utilized the pivot language approach to extracting phrasal paraphrases. Finally, the Viterbi decoding method was involved to generate question reformulations. The experimental results indicated that both the proposed phrasal paraphrase extraction and the question reformulation-based question retrieval methods outperformed the state-of-the-art methods significantly. It demonstrated the effectiveness of our question reformulation method.

Inspired by [46]–[48], in the future, we expect to combine multiple methods on paraphrase extraction in order to optimize the estimation of paraphrase probability and get better results. We plan to combine the monolingual and bilingual based paraphrase extraction methods by integrating the intermediate results of the above two methods. And then we can estimate the paraphrase probabilities by linearly combining the two generated paraphrase probabilities with different integration weights. Finally, we can obtain a new paraphrase ranking list through the jointly estimating of the candidate paraphrases generated by the above two methods.
