# Correction: Phrasal Paraphrase Based Question Reformulation for Archived Question Retrieval

**DOI:** 10.1371/annotation/4bb6b73b-b5bb-4143-9ec3-99c90b93f3ad

**Published:** 2013-09-23

**Authors:** Yu Zhang, Wei-Nan Zhang, Ke Lu, Rongrong Ji, Fanglin Wang, Ting Liu

Information was omitted from the Funding statement. The correct version of the Funding statement is available below.

"This work was supported by the Natural Science Foundation of China (Grant No. 61133012, 61073129, 61073126) and the Fundamental Research Funds for the Central Universities (No.2013121026<tel:2013121026> and No.2011121052) and Xiamen University 985 Project. The funders had no role in study design, data collection and analysis, decision to publish, or preparation of the manuscript." 

